# Next-generation sequencing based newborn screening and comparative analysis with MS/MS

**DOI:** 10.1186/s12887-024-04718-x

**Published:** 2024-04-01

**Authors:** Guosong Shen, Wenwen Li, Yaqin Zhang, Lyuyan Chen

**Affiliations:** 1 Medical Laboratory Center, Huzhou Maternity & Child Health Care Hospital, Huzhou, Zhejiang Province 313000 China; 2 Institut for Neuroscience, Technical University of Munich, 80802 Munich, Germany

**Keywords:** Newborn screening, Inherited metabolic diseases, Gene mutations, Next-generation sequencing, Tandem mass spectrometry

## Abstract

**Background:**

Newborn screening (NBS), such as tandem mass spectrometry (MS/MS), may yield false positive/negative results. Next-generation sequencing (NGS) has the potential to provide increased data output, efficiencies, and applications. This study aimed to analyze the types and distribution of pathogenic gene mutations in newborns in Huzhou, Zhejiang province, China and explore the applicability of NGS and MS/MS in NBS.

**Methods:**

Blood spot samples from 1263 newborns were collected. NGS was employed to screen for pathogenic variants in 542 disease-causing genes, and detected variants were validated using Sanger sequencing. Simultaneously, 26 inherited metabolic diseases (IMD) were screened using MS/MS. Positive or suspicious samples identified through MS/MS were cross-referenced with the results of NGS.

**Results:**

Among all newborns, 328 had no gene mutations detected. NGS revealed at least one gene mutation in 935 newborns, with a mutation rate of 74.0%. The top 5 genes were *FLG*, *GJB2*, *UGT1A1*, *USH2A*, and *DUOX2*. According to American College of Medical Genetics guidelines, gene mutations in 260 cases were classified as pathogenic or likely pathogenic mutation, with a positive rate of 20.6%. The top 5 genes were *UGT1A1*, *FLG*, *GJB2*, *MEFV*, and *G6PD*. MS/MS identified 18 positive or suspicious samples for IMD and 1245 negative samples. Verification of these cases by NGS results showed no pathogenic mutations, resulting in a false positive rate of 1.4% (18/1263).

**Conclusion:**

NBS using NGS technology broadened the range of diseases screened, and enhanced the accuracy of diagnoses in comparison to MS/MS for screening IMD. Combining NGS and biochemical screening would improve the efficiency of current NBS.

**Supplementary Information:**

The online version contains supplementary material available at 10.1186/s12887-024-04718-x.

## Introduction

Newborn screening (NBS) was defined as specialized examinations conducted during the neonatal period, to detect congenital and hereditary diseases which pose a significant threat to newborn health. This process aims to provide early diagnosis and treatment and prevent irreversible damage to the infant’s vital organs [1]. NBS plays a pivotal role in controlling birth defects and enhancing the quality of the newborn population. The establishment of NBS was based on early work in the management of phenylketonuria [2]. In the 1960s, with the introduction of screening for phenylketonuria, which is known as bacterial inhibition assay, mass biochemical testing of newborn babies was pioneered [3]. In 1990s, the adoption of tandem mass spectrometry (MS/MS) in newborn screening blood spots revolutionized NBS [4], by enabling rapidly expand the numbers of diseases screened and reducing costs and time for testing [2]. Currently, MS/MS is a commonly used technology for screening inherited metabolic diseases (IMD) in newborns. It involves the measurement of metabolic products, such as amino acids, fatty acids, and organic acids [4]. While researchers in various countries have made multifaceted efforts to enhance the positive predictive value of IMD screening using MS/MS [5, 6], the challenge of false negatives and false positives remains unresolved. With the advancements in Next-Generation Sequencing (NGS), gene-based screening methods for newborn disease screening have the potential to reduce false positive rates, enhance positive predictive values, and increase their clinical applicability [7, 8]. Moreover, NGS allows for screening of hundreds of single-gene diseases concurrently and is considered a feasible and cost-effective screening tool [9].

Motivated by these benefits and challenges, this study employed NGS and MS/MS technologies to conduct NBS on 1263 newborns in Huzhou, Zhejiang province, China. Our aim is to explore the mutation types and distribution of pathogenic gene mutations in newborns from the Huzhou region, and to examine the comparative utility of NGS and MS/MS technologies in NBS.

## Subjects and methods

### Participants

This study was conducted between October 2022 and January 2023 and involved 1263 newborns born at Huzhou Maternal and Child Health Hospital in the Huzhou, Zhejiang province, China. The number of newborns born at the Huzhou Maternal and Child Health Hospital constitutes more than half of the total number of newborns in this city. These newborns were screened within 48 to 72 h of birth. Of the total, there were 638 male infants and 625 female infants (gender ratio: 1.02: 1.00). Blood samples from the newborns were collected from heel blood, by obtaining at least two blood spots, each with a diameter of 8 mm, on filter paper within 48 to 72 h of birth. A total of 1263 dried blood spot samples were obtained. All participants underwent screening for IMD using NGS technology. Additionally, they were subjected to MS/MS screening. This research was approved by the Medical Ethics Committee of the Huzhou Maternal and Child Health Hospital, and informed consent was obtained from the parents of all newborns who involved in this study. Testing results would be informed and explained to parents of participants. For those identified as positive or suspicious positive, sample would be reassessed by the same initial screening procedure again. Once the second testing is still positive, a telephone follow-up would be initiated and conducted.

### NGS technology for genetic screening of inherited metabolic diseases

#### DNA extraction and Library Construction

After the collection of blood spot samples, genomic DNA was extracted using the Qiagen DNA Mini Kit (Qiagen, Shanghai, China). After DNA quality assessment, the genomic DNA was fragmented using the S220 Focused-ultrasonicator instrument (Covaris, Massachusetts, USA). Library construction was carried out using library preparation reagents from MyGenotics (Beijing, China), which included end repair, adapter ligation, and polymerase chain reaction (PCR) amplification.

#### NGS technology

For the capture of fragmented DNA libraries, a gene capture kit (MyGenostics Inc, Beijing, China) was employed. The application of NGS in this study was targeted gene sequencing. The selected genes were either known pathogenic genes or genes of interest which encompassed the following categories: 71 genes associated with clinically screened diseases, 145 genes recommended in the American College of Medical Genetics guidelines for screening, 249 genes from the list of rare diseases that are preventable and treatable, 223 genes from the National Rare Disease Directory, 81 genes related to diseases screened using MS/MS (including all the genetic metabolic disease genes screened by MS/MS in this study), and the top 94 genes from the Mygenostics database. After removing duplicates, a total of 601 genes were involved, covering 542 subtypes of diseases (spanning 13 major categories of genetic diseases affecting systems such as the skeletal, respiratory, and urinary systems) (Supplementary Table 1). The biotinylated capture probes were designed to include coding exons of all genes as well as 50 bp flanking regions upstream and downstream of the exons, with a probe length of 100 bp. The capture experiments were conducted following the manufacturer’s instructions. Purification of PCR products was carried out using SPRI beads (Beckman Coulter). Enriched libraries were subjected to paired-end sequencing on a DNBSEQ-T7 sequencer (DNBSEQ) with a read length of 150 bp.

#### Identifying pathogenic mutations

After sequencing quality control, read mapping to the human reference genome (GRCh37/hg19) was performed to detect variations in SNPs and InDels. The data were transformed into VCF format, and variant annotation was carried out using the ANNOVAR software (http://annovar.openbioinformatics.org/en/latest/). Annotations included data from multiple databases, such as the 1000 Genomes Project, ESP6500, dbSNP, EXAC, an internal database (Mygenotics), and HGMD. Variant pathogenicity was predicted using REVEL, SIFT, PolyPhen-2, MutationTaster, and GERP++. Copy number variations were analyzed using NGS data based on depth-based strategy. Pathogenicity analysis of the variants followed the guidelines of the American College of Medical Genetics and Genomics/Association for Molecular Pathology (ACMG/AMP) [10], and the expert guidelines provided by ClinGen (https://cspec.genome.network/cspec/ui/svi/) to assess the pathogenicity of the identified variants.

#### Verification of pathogenic mutations

For pathogenic sites, specific PCR primers were designed for PCR amplification, followed by sequencing analysis. The verification methods differed depending on the gene: (1) *UGT1A1*, *FLG*: Normal captured sequencing was performed. Sanger sequencing was used to validate variant results. (2) *HBA*, *HBB*: Custom probe design was employed for capture sequencing. Special bioinformatics methods were used for analysis, followed by validation using a fluorescent PCR melting curve method with a thalassemia gene testing kit for Mediterranean anemia. (3) *SMN1*: Special bioinformatics methods were applied for analysis, followed by validation using the corresponding Multiplex Ligation-dependent Probe Amplification assay kit.

### MS/MS screening for inherited metabolic diseases

MS/MS screening was conducted for 26 genetic metabolic diseases, including hyperphenylalaninemia (HPA). MS/MS utilized a non-derivatized assay kit for the quantification of various amino acids, carnitine, and succinylacetone (NeoBase™, PerkinElmer). It involved the measurement and assessment of the concentrations of amino acids, succinylacetone, free carnitine, and acylcarnitines in dried blood spots collected on filter paper from newborns. In cases where positive or suspected IMD results were observed, they were cross-referenced with NGS for verification.

## Results

### Screening results of NGS

#### Prevalence of top 5 gene mutation among participants

Among the 1263 newborns, 328 individuals showed no gene mutations, while 935 individuals had at least one gene mutation, resulting in a gene mutation rate of 74.0%. The top 5 genes with the highest mutation frequencies, were *FLG*, *GJB2*, *UGT1A1*, *USH2A*, and *DUOX2*, with mutation rates in the population of 11.5%, 11.1%, 10.0%, 7.3%, and 6.6%, respectively (Table 1).

**Table 1:** Prevalence of top 5 gene mutation among participants

TOP	Gene	Relevant Diseases /Phenotypes (Genetic Modes)	Number of Case with Mutations	Mutation Rate
1	*FLG*	1. Ichthyosis Vulgaris (AD, AR) 2. Hereditary Atopic Dermatitis Type 2 (-)	145	11.5%
2	*GJB2*	Autosomal Recessive Deafness Type 1 A (AR, AD)	141	11.1%
3	*UGT 1A1*	1. Familial Transient Neonatal Hyperbilirubinemia (AD, AR) 2. Bilirubin, Serum Levels, Quantitative Trait Locus 1; BILIQTL1 (-) 3. Crigler-Najjar Syndrome Type 1 (AR) 4. Crigler-Najjar Syndrome Type 2 (AR) 5. Gilbert Syndrome (AR)	126	10.0%
4	*USH2A*	1. Usher Syndrome Type IIA (AR) 2. Retinitis Pigmentosa Type 39 (AR)	92	7.3%
5	*DUOX2*	Thyroid Hormone Disorder Type 6 (AR)	83	6.6%

*Abbreviation* AR, Autosomal Recessive; AD, Autosomal Dominant

#### Prevalence in popular genes mutation among participants

Additionally, we presented the mutation prevalence of genes commonly used for screening (i.e. *HBA*, *SMN1*, *PAH*, *DMD* and *HBB*) in local area. Among 1263 newborns, the mutation rates for the *HBA*, *SMN1*, *PAH*, *DMD* and *HBB* genes were 5.0%, 3.3%, 2.1%, 1.9% and 1.2%, respectively (Table 2).

**Table 2:** Prevalence in popular genes mutation among participants

	Gene	Relevant Diseases /Phenotypes (Genetic Modes)	Number of Case with Mutations	Mutation Rate
1	*HBA*	Alpha-Thalassemia, also known as α-Thalassemia	63	5.0%
2	*SMN1*	Spinal Muscular Atrophy (AR)	42	3.3%
3	*PAH*	Phenylketonuria (AR)	26	2.1%
4	*DMD*	1. Duchenne Muscular Dystrophy (X-linked recessive) 2. Becker Muscular Dystrophy (XLR) 3. Dilated Cardiomyopathy Type 3B (XL)	24	1.9%
5	*HBB*	Beta-Thalassemia, also known as β-Thalassemia	15	1.2%

*Abbreviation* AR, Autosomal Recessive; AD, Autosomal Dominant; XLR: X-linked recessive; XL: X-linked

#### Prevalence of gene mutations classified as pathogenic or likely pathogenic mutations

According to the types of gene mutations, including homozygous mutations, compound heterozygous mutations, and hemizygous mutations, and the database indications of pathogenic or likely pathogenic mutations, 260 cases exhibited gene mutations classified as pathogenic or likely pathogenic mutation, resulting in a positive rate of 20.6% (260/1263). Among all cases (*n* = 260), the prevalence of top 5 gene (*UGT1A1*, *FLG*, *GJB2*, *MEFV* and *G6PD*) were 36.5%, 35.0%, 5.8%, 3.1%, 2.7%, respectively (Table 3). Among all participants (*n* = 1263), the prevalence of top 5 genes (*UGT1A1*, *FLG*, *GJB2*, *MEFV* and *G6PD*) were 7.5%, 7.2%, 1.2%, 0.6%, and 0.6%, respectively (Table 3).

**Table 3:** Prevalence of gene mutations classified as pathogenic or likely pathogenic

TOP	Gene	Relevant Diseases /Phenotypes (Genetic Modes)	Number of Case	Prevalence among all cases	Prevalence among all participants
1	*UGT1A1*	1. Familial Transient Neonatal Hyperbilirubinemia (AD, AR) 2. Bilirubin, Serum Levels, Quantitative Trait Locus 1; BILIQTL1(-) 3. Crigler-Najjar Syndrome Type 1 (AR) 4.Crigler-Najjar Syndrome Type 2 (AR) 5. Gilbert Syndrome (AR)	95	36.5%	7.5%
2	*FLG*	1. Ichthyosis Vulgaris (AD, AR) 2. Hereditary Atopic Dermatitis Type 2 (-)	91	35.0%	7.2%
3	GJB2	Autosomal Recessive Deafness Type 1 A (AR, AD)	15	5.8%	1.2%
4	*MEFV*	1. Familial Mediterranean Fever (AR) 2. Acute Febrile Neutrophilic Dermatosis; AFND (AD) 3. Autosomal Dominant Familial Mediterranean Fever (AD)	8	3.1%	0.6%
5	*G6PD*	Glucose-6-Phosphate Dehydrogenase Deficiency (XL)	7	2.7%	0.6%

*Abbreviation* AR, Autosomal Recessive; AD, Autosomal Dominant; XL: X-linke

#### Clinical phenotype of newborns with gene mutations classified as pathogenic or likely pathogenic mutation

Clinical phenotype data of newborns were collected among cases with gene mutations classified as pathogenic or likely pathogenic mutation (*n* = 260) within three months of birth (Supplementary Table 2). Newborns with mild physiological jaundice that naturally resolves without significant harm were not included. The results revealed that there were 22 cases (23.2%) of severe pathological jaundice among cases with pathogenic or likely pathogenic *UGT1A1* gene mutations (*n* = 95). 15 cases (16.5%) exhibited symptoms such as eczema, allergic dermatitis (mild), or atopic dermatitis among associated pathogenic or likely pathogenic gene mutations (*n* = 91). Among 7 cases with *G6PD* mutation abnormalities, 1 female infant with heterozygous mutations displayed a normal phenotype, while the remaining 6 male infants exhibited glucose-6-phosphate dehydrogenase deficiency.

### Screening results of MS/MS

In this study, MS/MS screening was conducted for 26 genetic metabolic diseases and a total of 18 cases with suspicious or positive screening results were identified. Subsequently, all 18 cases were confirmed by the results of NGS, and no pathogenic genes were found. Meanwhile, there were no participants screened as suspicious or positive cases by NGS for the 26 genetic metabolic diseases. Thus, the false positive rate and true negative rate 1.4% (18/1263) and 98.6% (1245/1263), respectively. The list of screened diseases and screening diagnostic results can be found in Table 4.

**Table 4:** Screening results for 1263 newborns for inherited metabolic disorders

Disease Names	Number of Suspected or Positive Cases	Number of Genetically Confirmed Cases
**Amino Acid Metabolism Disorders**:	9	0
Phenylketonuria	3	0
Cystinuria	2	0
Maple Syrup Urine Disease	-	-
Tyrosinemia	1	0
Citrullinemia Type I	-	-
Citrullinemia Type II	2	0
Arginemia	1	0
Ornithine Transcarbamylase Deficiency	-	-
**Organic Acid Metabolism Disorders**:	6	0
Glutaric Acidemia Type I	1	0
Methylmalonic Acidemia	1	0
Propionic Acidemia	-	-
Isovaleric Acidemia	1	0
Beta-Ketothiolase Deficiency	-	-
Glutaric Acidemia Type II	-	-
3-Methylglutaconyl-CoA Carboxylase Deficiency	-	-
3-Hydroxy-3-Methylglutaric Acidemia	1	0
Multiple Carboxylase Deficiency	1	0
Succinic Acidemia	1	0
**Organic Acid Metabolism Disorders**:	3	0
Very Long-Chain Acyl-CoA Dehydrogenase Deficiency	-	-
Long-Chain 3-Hydroxyacyl-CoA Dehydrogenase Deficiency	-	-
Trifunctional Protein Deficiency	1	0
Medium-Chain Acyl-CoA Dehydrogenase Deficiency	1	0
Short-Chain Acyl-CoA Dehydrogenase Deficiency	-	-
Carnitine Uptake Deficiency	-	-
Carnitine Palmitoyltransferase I Deficiency	1	0
Carnitine Palmitoyltransferase II Deficiency	-	-

## Discussion

NBS, as a crucial element of tertiary prevention for reducing birth defects, holds significant importance in reducing the risks associated with birth defects [11]. Various methods are employed for NBS, including newborn metabolic disease screening (such as congenital hypothyroidism, phenylketonuria, congenital adrenal hyperplasia, G6PD deficiency), hearing screening, fundus examination, and congenital heart disease blood oxygen saturation screening. While, these conventional methods for screening exists some limitations [12], including susceptibility to interference factors, leading to false positives or false negatives. Meanwhile, traditional methods provide only preliminary diagnoses, without precise disease typing. Consequently, efficient and accurate screening techniques and strategies are urgently needed.

The MS/MS screening technology used in this study demonstrates the high accuracy for some IMD, such as propionic acidemia [13] and HPA [14]. However, MS/MS screening is limited to specific types of IMD, and the scope of diseases screened is restricted. Clinical assessment typically relies on detecting metabolite concentrations or enzyme activity to diagnose disease. While enzyme activity testing has stringent sample conditions and may involve fluctuating changes in specific metabolic markers based on the health status of individuals [15]. Consequently, when using MS/MS analysis in NBS to screen for some certain diseases, there is an elevated risk of false positives, such as galactosemia [16], phenylketonuria type 1 [17], and isovaleric acidemia [18]. Additionally, some genetic metabolic diseases are not included in newborn biochemical screening due to the insensitivity or specificity of existing biochemical markers, for example, N-acetylglutamate synthase deficiency and carbamoyl phosphate synthetase 1 deficiency [19]. Although this study had a relatively small sample size for IMD screening, all follow-ups were conducted meticulously and our results revealed a false positive rate of 1.4% in MS/MS compared to NGS.

The newborn gene screening used in this study was NGS technology, which would provide some advantages such as faster sequencing, broader sequencing range, higher sensitivity, greater precision, and lower cost when compared to traditional sequencing methods (Sanger sequencing) [20]. NGS has been widely used in various fields, including cancer, prenatal screening, drug development, and the diagnosis of various genetic diseases [21, 22]. A pilot study conducted in Slovenia for screening selected inborn errors of metabolism, using tandem mass spectrometry followed by NGS, and indicated that the application NGS would improve the early and accurate identification of diseases [23]. Besides, the NGS technology has also been used to evaluate explore panels of genes and identify complex mechanisms of some pathogenesis, such as congenital hypothyroidism with dyshormonogenesis [24], and to help in the diagnosis of rare pediatric diseases [25]. Currently, the use of NGS technology for newborn genome screening is gradually gaining concerns in China [26]. This study covered a wide range of diseases, encompassing 13 major categories of genetic disorders, 542 disease subtypes, and the screening of 601 genes in newborn genome screening. Results were obtained within an average of one week after blood collection, underscoring the significant advantages and developmental potential of NGS technology for the management of birth defects.

This study identified two high-frequency pathogenic gene mutations. One is the *UGT1A1* gene, with 7.5% of pathogenic and likely pathogenic mutations accounted for among all participants. 22 cases of infants with severe pathological jaundice related to *UGT1A1* gene mutations were identified, with a 1.7% positivity rate among all participants. *UGT1A1* gene mutations are the molecular genetic basis for Gilbert syndrome (GS) and Crigler-Najjar syndrome (CNS) [27]. The *UGT1A1* gene encodes glucuronyltransferase, a critical enzyme in bilirubin metabolism responsible for converting unconjugated bilirubin into conjugated bilirubin [28]. Deficiency in this enzyme leads to congenital jaundice conditions known as GS and CNS. Clinical manifestations include elevated unconjugated bilirubin levels in newborns and persistent jaundice in adults. as confirmed in previous reports, such as by Yang Hui and colleagues [29], who detected *UGT1A1* gene mutations in 42 out of 61 severe jaundiced children. The most frequent *UGT1A1* gene mutation in this study was the c.211G > A (exon1, NM_000463.3) site, resulting in the amino acid change p.Gly71Arg, which is a missense mutation. According to ACMG guidelines, this mutation was classified as uncertain clinical significance (Uncertain BP4_Moderate). *UGT1A1* gene mutations exhibit polymorphism, with different populations in various regions having specific mutation types [30]. The c.211G > A (exon1, NM_000463.3) mutation site is the most common mutation in Asian populations [31]. Another high-frequency mutation site, c.1091 C > T (exon4, NM_000463.3), has been reported in jaundiced children in other Asian regions and has been demonstrated to cause reduced UGT1 enzyme activity and clinical observations related to GS [32]. Its relevance to neonatal jaundice requires further investigation with a larger sample size.

The second high-frequency pathogenic gene is the *FLG* gene, with pathogenic and likely pathogenic mutations accounting for 7.2% of all participants. 15 cases with eczema, mild allergic dermatitis, or atopic dermatitis were identified, comprising 1.2% positivity rate in all participants. The *FLG* gene, also known as the filaggrin gene, encodes filaggrin, a key component of the stratum corneum. Gene mutations can result in reduced or absent natural moisturizing factors, impaired stratum corneum function, and associated skin conditions. A study by Qian Qiufang et al. [33] in Shanghai, China investigated *FLG* gene mutations in over 300 adolescents, revealing associations between *FLG* gene mutations and atopic dermatitis, ichthyosis, asthma, and allergic rhinitis. A European study indicated that *FLG* gene mutation rates were 26.7% in children with atopic dermatitis and 14.4% in healthy children [34], with a correlation to concurrent asthma [35]. Additionally, the most frequent mutation site in the *FLG* gene in this study was c.12,064 A > T (exon 3, NM_002016.2), leading to an amino acid change p.Lys4022Ter, a nonsense mutation. According to ACMG guidelines, this mutation was classified as a pathogenic variant.

Early screening and diagnosis of gene mutations in newborns allows for prompt management, preventing adverse outcomes associated with these conditions. Prompt identification of *UGT1A1* gene mutations help to address jaundice and prevent severe conditions like CNS. It also facilitates genetic counseling, reducing the psychological burden on families. Similarly, early detection of *FLG* gene mutations helps alleviate discomfort and complications associated with skin conditions, improving the long-term health of affected infants. Our study also investigated several gene mutations commonly used for screening, such as Mediterranean anemia-related genes *HBA* and *HBB*, progressive muscular dystrophy-related gene *DMD*, spinal muscular atrophy-related gene *SMN1*, and phenylketonuria-related gene *PAH*, confirming their high mutation rates and clinical value. Meanwhile, this study provided insights into the genetic mutation profile of newborn in the Huzhou, Zhejiang province, China, allowing us to gain a preliminary understanding of the hereditary diseases with high prevalence among local newborns. With this knowledge, we can further effectively guide efforts in birth defect prevention and control.

However, there are still some limitations that need to be noted. First, the participants in this study were not selected randomly but were included based on the principle of voluntary participation by parents. Meanwhile, this study was conducted only in one hospital. Although this hospital accounted for more than half of the newborn births in Huzhou city, we have to acknowledge that there is a certain selection bias in this population. Second, this study was conducted in the northern region of Zhejiang province in China; thus, our findings may not be generalizable to children in other regions and countries.

## Conclusion

Through this study, NGS technology was found to reduce the rates of false positives in comparison to MS/MS for screening IMD, substantially enhancing the accuracy of diagnoses in NBS. It also expands the scope of genetic disease screening, leading to higher screening efficiency. In summary, our research underscored the efficiency of combining NGS screening with biochemical screening in NBS.

### Electronic supplementary material

Below is the link to the electronic supplementary material.

Supplementary Material 1

## Data Availability

The dataset have been deposited in ClinVAR (accession numbers: SCV004800908 -SCV004800929).

## Abbreviations

NBS: Newborn screening
MS/MS: Tandem mass spectrometry
IMD: Inherited metabolic diseases
NGS: Next-Generation Sequencing
HPA: Hyperphenylalaninemia
PCR: Polymerase chain reaction
ACMG: American College of Medical Genetics
AMP: Association for Molecular Pathology
