# Supplementary material for: Next-generation sequencing based newborn screening and comparative analysis with MS/MS
Source: BMC Pediatr. 2024 Apr 1;24:230. doi: 10.1186/s12887-024-04718-x (PMC10985934; doi:10.1186/s12887-024-04718-x)
Supplement: Supplementary file 1 — Supplementary Material 1 [file 12887_2024_4718_MOESM1_ESM.docx]

**Supplementary table 1.** The details of 601 genes and 542 disease subtypes.

| **Gene** | **Disease** | **The mode of inheritance** |
| --- | --- | --- |
| *ABCA4* | Stargardt disease type 1, Retinitis pigmentosa type 19, Age-related macular degeneration type 2, Cone-rod dystrophy type 3 | Autosomal recessive |
| *ABCB11* | Progressive familial intrahepatic cholestasis type 2 | Autosomal recessive |
| *ABCB4* | Progressive familial intrahepatic cholestasis type 3 | Autosomal recessive |
| *ABCC8* | Hyperinsulinemic hypoglycemia | Autosomal dominant |
| *ABCD1* | X-linked adrenoleukodystrophy | X-linked recessive |
| *ABCG5* | Sitosterolemia | Autosomal recessive |
| *ABCG8* | Sitosterolemia | Autosomal recessive |
| *ACADM* | Medium-chain acyl-CoA dehydrogenase deficiency | Autosomal recessive |
| *ACADS* | Short-chain acyl-CoA dehydrogenase deficiency | Autosomal recessive |
| *ACADVL* | Very long-chain acyl-CoA dehydrogenase deficiency | Autosomal recessive |
| *ACAT1* | Alpha-methylacetoacetic aciduria | Autosomal recessive |
| *ADA* | Adenosine deaminase deficiency leading to autosomal recessive severe combined immunodeficiency | Autosomal recessive, Somatic mosaicism |
| *AGA* | Aspartylglucosaminuria | Autosomal recessive |
| *AGL* | Glycogen storage disease type III | Autosomal recessive |
| *ALDOB* | Hereditary fructose intolerance | Autosomal recessive |
| *ALPL* | Hypophosphatasia, infantile and adult forms | Autosomal recessive |
| *AMT* | Glycine encephalopathy | Autosomal recessive |
| *ARSA* | Metachromatic leukodystrophy | Autosomal recessive |
| *ARSB* | Mucopolysaccharidosis type VI | Autosomal recessive |
| *ASL* | Argininosuccinic aciduria | Autosomal recessive |
| *ASS1* | Classical citrullinemia | Autosomal recessive |
| *ATM* | Ataxia telangiectasia | Autosomal recessive |
| *ATP7B* | Wilson disease | Autosomal recessive |
| *BBS1* | Bardet-Biedl syndrome types 1 | DR, Autosomal recessive |
| *BBS2* | Bardet-Biedl syndrome types 2 | Autosomal recessive |
| *BCKDHA* | Maple syrup urine disease | Autosomal recessive |
| *BCKDHB* | Maple syrup urine disease | Autosomal recessive |
| *BTD* | Biotinidase deficiency | Autosomal recessive |
| *BTK* | X-linked agammaglobulinemia type 1 | X-linked recessive |
| *CAPN3* | Limb-girdle muscular dystrophy type 2A | Autosomal recessive |
| *CBS* | Homocystinuria due to cystathionine beta-synthase deficiency | Autosomal recessive |
| *CEP290* | Bardet-Biedl syndrome type 14 | Autosomal recessive |
| *CFTR* | Cystic fibrosis; bronchiectasis with or without elevated sweat chloride, type 1; bronchiectasis with or without elevated sweat chloride, type 1 | Autosomal recessive |
| *CHRNE* | Congenital myasthenic syndrome type 4A | Autosomal dominant, Autosomal recessive |
| *CLN3* | Neuronal ceroid lipofuscinosis types 3 | Autosomal recessive |
| *CLN5* | Neuronal ceroid lipofuscinosis types 5 | Autosomal recessive |
| *CLN6* | Neuronal ceroid lipofuscinosis types 4A | Autosomal recessive |
| *COL4A3* | Autosomal dominant Alport syndrome | Autosomal dominant |
| *COL4A4* | Autosomal recessive Alport syndrome | Autosomal recessive |
| *COL7A1* | Autosomal recessive dystrophic epidermolysis bullosa | Autosomal recessive |
| *CPS1* | Carbamoyl phosphate synthetase I deficiency | Autosomal recessive |
| *CPT2* | Neonatal lethal carnitine palmitoyltransferase II deficiency | Autosomal recessive |
| *CTNS* | Nephropathic cystinosis | Autosomal recessive |
| *CYP11B1* | 11-beta-hydroxylase deficiency | Autosomal recessive |
| *CYP4V2* | Bietti's crystalline corneoretinal dystrophy | Autosomal recessive |
| *DBT* | Maple syrup urine disease | Autosomal recessive |
| *DLD* | Maple syrup urine disease types 3 | Autosomal recessive |
| *DMD* | Duchenne and Becker muscular dystrophy | X-linked recessive |
| *DYSF* | Miyoshi myopathy; Limb-girdle muscular dystrophy type 2B; Distal myopathy with anterior tibial onset | Autosomal recessive |
| *ETFA* | Glutaric acidemia type II | Autosomal recessive |
| *ETFB* | Glutaric acidemia type II | Autosomal recessive |
| *ETFDH* | Glutaric acidemia type II | Autosomal recessive |
| *F9* | Hemophilia B | X-linked recessive |
| *FAH* | Tyrosinemia type I | Autosomal recessive |
| *FANCA* | Fanconi anemia group A | Autosomal recessive |
| *G6PC* | Glycogen storage disease type Ia | Autosomal recessive |
| *GAA* | Glycogen storage disease type II | Autosomal recessive |
| *GALC* | Krabbe disease | Autosomal recessive |
| *GALK1* | Galactokinase deficiency | Autosomal recessive |
| *GALNS* | Mucopolysaccharidosis type 4A | Autosomal recessive |
| *GALT* | Galactosemia | Autosomal recessive |
| *GBA* | Gaucher disease types 1, 2, 3C, and perinatal lethal | Autosomal recessive |
| *GBE1* | Glycogen storage disease type IV | Autosomal recessive |
| *GCDH* | Glutaric acidemia type I | Autosomal recessive |
| *GJB2* | Vohwinkel syndrome; autosomal dominant hereditary deafness 3A; autosomal dominant keratitis-ichthyosis-deafness syndrome; autosomal recessive deafness 1A; harlequin ichthyosis with deafness (HID syndrome); palmoplantar keratoderma with deafness; and knuckle pads-white nails-sensorineural deafness syndrome | Autosomal dominant |
| *GLA* | Fabry disease | X-linked |
| *GLB1* | GM1 gangliosidosis, Mucopolysaccharidosis type IVB | Autosomal recessive |
| *GLDC* | Glycine encephalopathy | Autosomal recessive |
| *GNPTAB* | Mucolipidosis types III alpha/beta | Autosomal recessive |
| *GNS* | Mucopolysaccharidosis type IIID | Autosomal recessive |
| *GPR143* | X-linked congenital nystagmus type 6; Leber's congenital amaurosis type 1 | Unknown |
| *HADH* | 3-Hydroxyacyl-CoA dehydrogenase deficiency | Autosomal recessive |
| *HBA1* | Alpha and beta thalassemias | Unknown |
| *HBA2* | Alpha and beta thalassemias | Unknown |
| *HBB* | Beta-thalassemia; sickle cell anemia. | Unknown、Autosomal recessive |
| *HEXA* | Tay-Sachs disease | Autosomal recessive |
| *HEXB* | Sandhoff disease | Autosomal recessive |
| *HGSNAT* | Mucopolysaccharidosis type IIIC | Autosomal recessive |
| *HLCS* | Holocarboxylase synthetase deficiency | Autosomal recessive |
| *IDS* | Mucopolysaccharidosis type II | X-linked recessive |
| *IDUA* | Mucopolysaccharidosis Ih/s type; Mucopolysaccharidosis Ih type; Mucopolysaccharidosis Is type | Autosomal recessive |
| *IL2RG* | X-linked severe combined immunodeficiency | X-linked recessive |
| *IVD* | Isovaleric acidemia | Autosomal recessive |
| *KCNJ11* | Familial hyperinsulinism type 2 | Autosomal recessive |
| *LIPA* | Lysosomal acid lipase deficiency | Autosomal recessive |
| *LRMDA* | Vitiligo | - |
| *LRP5* | Polycystic liver disease type 4 with or without kidney cysts | Autosomal dominant |
| *MAN2B1* | Alpha-mannosidosis | Autosomal recessive |
| *MCCC1* | 3-Methylcrotonyl-CoA carboxylase deficiency type 1 | Autosomal recessive |
| *MCCC2* | 3-Methylcrotonyl-CoA carboxylase deficiency type 2 | Autosomal recessive |
| *MCEE* | Methylmalonyl-CoA isomerase deficiency | Autosomal recessive |
| *MCOLN1* | Type 4 mucopolysaccharidosis | Autosomal recessive |
| *MEFV* | Autosomal dominant familial Mediterranean fever | Autosomal dominant |
| *MFSD8* | Neuronal ceroid lipofuscinosis type 7 | Autosomal recessive |
| *MKS1* | Bardet-Biedl syndrome type 13 | Autosomal recessive |
| *MMAA* | Methylmalonic aciduria cblA type | Autosomal recessive |
| *MMAB* | Methylmalonic aciduria cblB type | Autosomal recessive |
| *MMACHC* | Methylmalonic acidemia with homocystinuria cblC type | Autosomal recessive |
| *MMADHC* | Methylmalonic acidemia with homocystinuria cblD type | Autosomal recessive |
| *MOCS1* | Molybdenum cofactor deficiency complementation group A | Autosomal recessive |
| *MTHFR* | Hyperhomocysteinemia | AR |
| *MTR* | Homocystinuria due to cystathionine beta-synthase deficiency type 1g | Autosomal recessive |
| *MT-RNR1* | Aminoglycoside-induced deafness | MT |
| *MTRR* | Homocystinuria due to cystathionine beta-synthase deficiency type 1e | Autosomal recessive |
| *MUT* | Methylmalonic aciduria due to methylmalonyl-CoA mutase deficiency | Autosomal recessive |
| *NAGLU* | Type 3B mucopolysaccharidosis | Autosomal recessive |
| *NPC1* | Niemann-Pick disease type C1 | Autosomal recessive |
| *NPC2* | Niemann-Pick disease type C2 | Autosomal recessive |
| *NPHS1* | Nephrotic syndrome type 1 | Autosomal recessive |
| *NR0B1* | Congenital adrenal hypoplasia | X-linked recessive |
| *OCA2* | Oculocutaneous albinism type 2 | Autosomal recessive |
| *OTC* | Hyperammonemia due to ornithine carbamoyltransferase deficiency | X-linked recessive |
| *PAH* | Phenylketonuria | Autosomal recessive |
| *PCCA* | Propionic acidemia | Autosomal recessive |
| *PCCB* | Propionyl-CoA carboxylase deficiency, ketotic glycineemia |  |
| *PHKA2* | Glycogen storage disease type IXa1 | X-linked recessive |
| *PKHD1* | Autosomal dominant polycystic kidney disease | Autosomal recessive |
| *PMM2* | Congenital glycosylation disorder type Ia | Autosomal recessive |
| *PPT1* | Neuronal ceroid lipofuscinosis type 1 | Autosomal recessive |
| *PRF1* | Familial hemophagocytic lymphohistiocytosis type 2 | Autosomal recessive |
| *PTS* | Tetrahydrobiopterin deficiency type A | Autosomal recessive |
| *PYGL* | Glycogen storage disease type VI | Autosomal recessive |
| *RAG1* | Severe combined immunodeficiency with α/βT-cell deficiency, γ/δT-cell expansion, severe cytomegalovirus infection with autoimmune disease, autosomal recessive T-negative B-negative NK-positive severe combined immunodeficiency | Autosomal recessive |
| *RAG2* | Autosomal recessive T-negative B-negative NK-positive severe combined immunodeficiency | Autosomal recessive |
| *RMRP* | Chondrodysplasia punctata | Autosomal recessive |
| *RS1* | X-linked retinoschisis | X-linked recessive |
| *SGSH* | Mucopolysaccharidosis type 3A | Autosomal recessive |
| *SLC12A3* | Gitelman syndrome | Autosomal recessive |
| *SLC22A5* | Primary systemic carnitine deficiency | Autosomal recessive |
| *SLC24A5* | Oculocutaneous albinism type 6 | Autosomal recessive |
| *SLC26A2* | Osteogenesis imperfecta type 2 | Autosomal recessive |
| *SLC26A4* | Pendred syndrome, autosomal recessive deafness type 6 with vestibular aqueduct dilatation | Autosomal recessive |
| *SLC37A4* | Glycogen storage disease type 1b | Autosomal recessive |
| *SLC45A2* | Oculocutaneous pigmentary anomalies type 5, oculocutaneous albinism type 4 | Autosomal recessive |
| *SLC6A8* | Cerebral creatine deficiency syndrome 1 |  |
| *SMN1* | Spinal muscular atrophy type 1, type 2, type 3, type 4 | Autosomal recessive |
| *SMPD1* | Niemann-Pick disease type B, type A | Autosomal recessive |
| *STX11* | Familial hemophagocytic lymphohistiocytosis type 4 | Autosomal recessive |
| *STXBP2* | Familial hemophagocytic lymphohistiocytosis type 5 | Unknown |
| *TCIRG1* | Autosomal recessive osteopetrosis type 1 | Autosomal recessive |
| *TGM1* | Ichthyosis vulgaris | Autosomal recessive |
| *TPP1* | Neuronal ceroid lipofuscinosis type 2 | Autosomal recessive |
| *TYR* | Oculocutaneous pigmentary anomalies type 3, oculocutaneous albinism associated with sensorineural deafness, oculocutaneous albinism type 1A, oculocutaneous albinism type 1B | Unknown、Autosomal dominant |
| *TYRP1* | Oculocutaneous pigmentary anomalies type 11, oculocutaneous albinism type 3 | Unknown、Autosomal recessive |
| *UGT1A1* | Gilbert syndrome | Autosomal recessive |
| *UNC13D* | Familial hemophagocytic lymphohistiocytosis type 3 | Unknown |
| *USH2A* | Usher syndrome IIA, retinitis pigmentosa type 39 | Autosomal recessive |
| *WAS* | Wiskott-Aldrich syndrome type 1 | X-linked recessive |
| *ABCA1* | Familial hypercholesterolemia | Autosomal dominant |
| *ABCB7* | Microcytic anemia with iron overload |  |
| *ABCC2* | Dubin-Johnson syndrome | Autosomal recessive |
| *ABCD3* | Congenital bile acid synthesis defect type 5 | Autosomal recessive |
| *ABCD4* | Methylmalonic acidemia with homocystinuria cblJ type | Autosomal recessive |
| *ACAD8* | Isovaleryl-CoA dehydrogenase deficiency (IBDH) | Unknown |
| *ACADSB* | 2-Methylbutyrylglycinuria | Autosomal recessive |
| *ACD* | Autosomal dominant congenital ichthyosis type 6/7 | Autosomal dominant, Autosomal recessive |
| *ACOX2* | Congenital bile acid synthesis defect type 6 | Autosomal recessive |
| *ACSF3* | Combined 2-methyl-3-hydroxybutyric aciduria and 2-methylbutyric aciduria | Unknown |
| *ACTC1* | Dilated cardiomyopathy type 1R | Autosomal dominant |
| *ACVR1* | Progressive osseous heteroplasia | Autosomal dominant |
| *ACVRL1* | Hereditary hemorrhagic telangiectasia type 2 | Autosomal dominant |
| *ADAMTS13* | Congenital thrombocytopenic purpura | Autosomal recessive |
| *ADK* | Hyperphenylalaninemia due to adenosine kinase deficiency | Autosomal recessive |
| *AGRN* | Congenital myasthenic syndrome type 8 | Autosomal recessive |
| *AHCY* | Hyperphenylalaninemia with S-adenosylhomocysteine hydrolase deficiency | Autosomal recessive |
| *AKR1D1* | Congenital bile acid synthesis defect type 2 | Autosomal recessive |
| *ALAS2* | Microcytic anemia with iron overload type 1 | X-linked recessive |
| *ALDH4A1* | Hyperprolinemia type 2 | Autosomal recessive |
| *ALDH6A1* | Methylmalonic semialdehyde dehydrogenase deficiency | Autosomal recessive |
| *ALDH7A1* | Pyridoxine-dependent seizures | Autosomal recessive |
| *ALDOA* | Glycogen storage disease type XII | Autosomal recessive |
| *ALG14* | Congenital myasthenic syndrome type 15 | Autosomal recessive |
| *ALG2* | Congenital myasthenic syndrome type 14 | Autosomal recessive |
| *AMACR* | Congenital bile acid synthesis defect type 4 | Autosomal recessive |
| *ANK1* | Spherocytosis type 1 | Autosomal dominant |
| *ANOS1* | Kallmann syndrome type 1 | X-linked recessive |
| *ANXA11* | Amyotrophic lateral sclerosis 23 | Autosomal dominant |
| *APEX1* | Nijmegen breakage syndrome | - |
| *APOB* | Autosomal dominant hypercholesterolemia type B, familial hypercholesterolemia type 1 | Autosomal dominant |
| *APOE* | Alzheimer's disease type 2 | Autosomal dominant |
| *AR* | Kennedy disease | X-linked recessive |
| *ARG1* | Hyperornithinemia | Autosomal recessive |
| *ASCL1* | Congenital central hypoventilation syndrome | Autosomal dominant |
| *ATP13A2* | Kufor-Rakeb syndrome | Autosomal recessive |
| *ATXN2* | Spinocerebellar ataxia type 2 | Autosomal dominant |
| *AUH* | 3-Methylglutaconic aciduria type 1 | Autosomal recessive |
| *AVPR2* | X-linked nephrogenic diabetes insipidus, antidiuretic hormone resistance syndrome | X-linked recessive |
| *BAX* | T-cell acute lymphoblastic leukemia, somatic mutation | - |
| *BBS10* | Bardet-Biedl syndrome type 10 | Autosomal recessive |
| *BBS12* | Bardet-Biedl syndrome type 12 | Autosomal recessive |
| *BCAT1* | Branched-chain amino acid metabolism disorder: hyperleucine, hyperisoleucine, hypervaline | - |
| *BCAT2* | Branched-chain amino acid metabolism disorder: hyperleucine, hyperisoleucine, hypervaline | - |
| *BCR* | T-cell acute lymphoblastic leukemia, somatic mutation | - |
| *BLK* | Late-onset diabetes type 11 | Autosomal dominant |
| *BMPR2* | Idiopathic pulmonary arterial hypertension | Autosomal dominant |
| *BRAF* | Nijmegen syndrome type 7 | Autosomal dominant |
| *BSND* | Bartter syndrome type 4A | Autosomal recessive |
| *C15orf41* | Congenital erythropoietic porphyria type 1b | Autosomal recessive |
| *C3* | Complement C3 deficiency | Autosomal recessive |
| *CA2* | Osteopetrosis with renal tubular acidosis | Autosomal recessive |
| *CACNA1D* | Primary hyperaldosteronism (with neurofibromatosis) | Autosomal dominant |
| *CACNA1H* | Childhood absence epilepsy susceptibility 6 | - |
| *CALR* | Myelofibrosis, somatic mutation | SMu |
| *CARD11* | B-cell proliferation with NFKB and T-cell anergy | Autosomal dominant |
| *CD3D* | Immunodeficiency type 19 | Autosomal recessive |
| *CD40LG* | X-linked hyper-IgM syndrome | X-linked recessive |
| *CD46* | Hemolytic uremic syndrome susceptibility type 2 | Autosomal dominant, Autosomal recessive |
| *CDAN1* | Congenital dyserythropoietic anemia type 1a | Autosomal recessive |
| *CEL* | Late-onset type 8 diabetes mellitus | Autosomal dominant |
| *CFB* | Hemolytic uremic syndrome susceptibility type 4 | Autosomal dominant |
| *CFH* | Complement factor H deficiency syndrome | Autosomal dominant, Autosomal recessive |
| *CFHR1* | Hemolytic uremic syndrome susceptibility type 1 | Autosomal dominant, Autosomal recessive |
| *CFHR3* | Hemolytic uremic syndrome susceptibility type 1 | Autosomal dominant, Autosomal recessive |
| *CFI* | Hemolytic uremic syndrome susceptibility type 3 | Autosomal dominant |
| *CHAT* | Congenital myasthenic syndrome type 6 | Autosomal recessive |
| *CHD7* | Kallmann syndrome type 5 | Autosomal dominant, Autosomal recessive |
| *CHRNA1* | Congenital myasthenic syndrome type 1A | Autosomal dominant |
| *CHRNB1* | Congenital myasthenic syndrome type 2A | Autosomal dominant |
| *CHRND* | Multiple self-healing squamous epithelioma syndrome | Autosomal recessive |
| *CITED2* | Ventricular septal defect type 2 | Autosomal dominant |
| *CLCN5* | Dent disease type 1, X-linked recessive hypophosphatemic rickets, low molecular weight proteinuria, hypercalciuria and nephrocalcinosis, renal tubular acidosis type I | X-linked recessive |
| *CLCN7* | Autosomal dominant osteopetrosis type 2 | Autosomal dominant |
| *CLCNKA* | Bartter syndrome type 4B due to double gene mutation | DR |
| *CLCNKB* | Bartter syndrome type 4B due to double gene mutation | DR |
| *CLDN16* | Renal hypomagnesemia type 3 | Autosomal recessive |
| *CLDN19* | Renal hypomagnesemia type 5 | Autosomal recessive |
| *CLN8* | Neuronal ceroid lipofuscinosis type 8 | Autosomal recessive |
| *CLTCL1* | DiGeorge syndrome |  |
| *CNNM2* | Hypomagnesemia, seizures and mental retardation, renal hypomagnesemia type 6 | Autosomal dominant, Autosomal recessive |
| *COL13A1* | Congenital myasthenic syndrome type 19 | Autosomal recessive |
| *COL1A1* | Osteogenesis imperfecta type 1 | Autosomal dominant |
| *COL1A2* | Osteogenesis imperfecta type 2 | Autosomal dominant |
| *COL4A5* | X-linked Alport syndrome | X-linked dominant |
| *COL9A1* | Stickler syndrome type 4 | Autosomal recessive |
| *COL9A2* | Hereditary multiple exostoses type 2 | Autosomal dominant |
| *COL9A3* | Hereditary multiple exostoses type 3 | Autosomal dominant |
| *COLQ* | Congenital myasthenic syndrome type 5 | Autosomal recessive |
| *COMP* | Pseudoachondroplasia | Autosomal dominant |
| *CPT1A* | Carnitine palmitoyltransferase 1 deficiency | Autosomal recessive |
| *CTH* | Cystathioninuria | Autosomal recessive |
| *CTNNA3* | Arrhythmogenic right ventricular dysplasia/cardiomyopathy type 13 | Autosomal dominant |
| *CTSD* | Neuronal ceroid lipofuscinosis type 10 | Autosomal recessive |
| *CTSF* | Neuronal ceroid lipofuscinosis type 13 | Autosomal recessive |
| *CYB5A* | High-affinity hemoglobinopathy type 4 | Autosomal recessive |
| *CYB5R3* | High-affinity hemoglobinopathy due to erythrocyte reductase deficiency | Autosomal recessive |
| *CYBB* | Immunodeficiency type 34 | X-linked recessive |
| *CYP17A1* | 17-α-hydroxylase deficiency congenital adrenal hyperplasia | Autosomal recessive |
| *CYP21A2* | 21-hydroxylase deficiency leading to congenital adrenal hyperplasia | Autosomal recessive |
| *CYP7B1* | Congenital bile acid synthesis defect type 3 | Autosomal recessive |
| *DAO* | Schizophrenia susceptibility type 12 | Autosomal dominant |
| *DCLRE1C* | Severe combined immunodeficiency with radiation sensitivity | Autosomal recessive |
| *DEPDC5* | Familial focal epilepsy with variable foci | Autosomal dominant |
| *DGCR2* | DiGeorge syndrome |  |
| *DKC1* | X-linked congenital ichthyosis type 6 | X-linked recessive |
| *DMP1* | Autosomal recessive hypophosphatemic rickets type 1 | Autosomal recessive |
| *DNAJC12* | Mild hyperphenylalaninemia, non-BH4-deficient type | Autosomal recessive |
| *DNAJC19* | 3-Methylglutaconic aciduria type 5 | Autosomal recessive |
| *DNAJC5* | Neuronal ceroid lipofuscinosis type 4 | Autosomal dominant |
| *DNM1L* | Mitochondrial peroxisomal biogenesis defect lethal encephalopathy | Autosomal dominant, Autosomal recessive |
| *DOCK8* | Hyper-IgE syndrome | Autosomal recessive |
| *DOK7* | Motor inability deformity sequence | Autosomal recessive |
| *DPAGT1* | Congenital glycosylation disorder type Ij | Autosomal recessive |
| *DSC2* | Arrhythmogenic right ventricular dysplasia/cardiomyopathy type 11 | Autosomal dominant, Autosomal recessive |
| *DSP* | Dilated cardiomyopathy with woolly hair, keratoderma, and tooth agenesis | Autosomal dominant |
| *DUOX2* | Thyroid hormone secretion disorder type 6 | Autosomal recessive |
| *DUOXA2* | Thyroid hormone secretion disorder type 5 | Autosomal recessive |
| *EDN3* | Von Willebrand disease type 4B | Autosomal dominant, Autosomal recessive |
| *EFL1* | Shwachman-Diamond syndrome type 2 | Autosomal recessive |
| *EGF* | Renal hypomagnesemia type 4 | Autosomal recessive |
| *EIF2AK3* | Multiple epiphyseal dysplasia with early-onset diabetes | Autosomal recessive |
| *ELANE* | Severe congenital neutropenia type 1 | Autosomal dominant |
| *ELN* | Williams syndrome | Autosomal dominant |
| *ELP3* | Amyotrophic lateral sclerosis | Autosomal dominant |
| *ENO3* | Glycogen storage disease type XIII | Autosomal recessive |
| *ENPP1* | Autosomal recessive hypophosphatemic rickets type 2 | Autosomal recessive |
| *EPM2A* | Hereditary deafness | Autosomal recessive |
| *ETHE1* | Ethylmalonic encephalopathy | Autosomal recessive |
| *F8* | Hemophilia A | X-linked recessive |
| *FBN1* | Marfan syndrome | Autosomal dominant |
| *FBP1* | Fructose-1,6-bisphosphatase deficiency | Autosomal recessive |
| *FBP2* | Glycogen storage disease | - |
| *FCGR2A* | Cystic fibrosis | Autosomal recessive |
| *FGA* | Congenital afibrinogenemia | Autosomal recessive |
| *FGB* | Congenital afibrinogenemia | Autosomal recessive |
| *FGF23* | Autosomal dominant hypophosphatemic rickets | Autosomal dominant |
| *FGFR1* | Kallmann syndrome type 2 | Autosomal dominant, Autosomal recessive |
| *FGG* | Congenital dysfibrinogenemia | Autosomal dominant |
| *FHL1* | X-linked scapuloperoneal myopathy, X-linked myopathy with muscle atrophy, X-linked dominant scapuloperoneal myopathy, X-linked severe congenital muscular dystrophy type 1A with onset in infancy or early childhood | X-linked |
| *FKBP10* | Osteogenesis imperfecta type 11 | Autosomal recessive |
| *FKBP6* | Williams syndrome | Autosomal dominant |
| *FLG* | Ichthyosis vulgaris | Autosomal dominant |
| *FLT3* | T-cell acute lymphoblastic leukemia, somatic mutation | - |
| *FOXE1* | Genes related to glycogen metabolism pathways | Autosomal recessive |
| *FTL* | High iron proteinemia cataract syndrome | Autosomal dominant |
| *FUCA1* | I-cell disease | Autosomal recessive |
| *FXYD2* | Renal hypomagnesemia type 2 | Autosomal dominant |
| *FZD4* | Familial exudative vitreoretinopathy type 1 | Autosomal dominant |
| *G6PC3* | Severe congenital neutropenia type 4 | Autosomal recessive |
| *G6PD* | Fabry disease | Autosomal dominant |
| *GALE* | Galactose epimerase deficiency | Autosomal recessive |
| *GAMT* | Guanidinoacetate methyltransferase deficiency | Autosomal recessive |
| *GATA1* | Thrombocytopenia with beta-thalassemia | X-linked recessive |
| *GATA4* | Testicular anomalies with or without congenital heart disease, Fallot tetralogy, ventricular septal defect type 2, atrioventricular septal defect type 4, ventricular septal defect type 1 | Autosomal dominant |
| *GATM* | Cerebral creatine deficiency syndrome 3 | Autosomal recessive |
| *GCH1* | GTP cyclohydrolase 1-deficient dopa-responsive dystonia, tetrahydrobiopterin deficiency type B | Autosomal dominant, Autosomal recessive |
| *GCK* | Late-onset type 2 diabetes mellitus | Autosomal dominant |
| *GCM2* | Hyperparathyroidism type 4 | Autosomal dominant |
| *GCSH* | Glutamic acidemia | Autosomal recessive |
| *GDNF* | Pheochromocytoma | Autosomal dominant |
| *GFM1* | Combined oxidative phosphorylation deficiency type 1 | Autosomal recessive |
| *GFPT1* | Congenital myasthenic syndrome type 12 | Autosomal recessive |
| *GHR* | Partial growth hormone insensitivity | Autosomal recessive |
| *GJB3* | Autosomal dominant nonsyndromic deafness type 2B, autosomal recessive nonsyndromic deafness type 1A, progressive variably pigmented macular hypomelanosis | Autosomal dominant |
| *GLIS3* | Neonatal diabetes mellitus with congenital hypothyroidism | Autosomal recessive |
| *GLRX5* | Congenital sideroblastic anemia type 3 | Autosomal recessive |
| *GLUD1* | Familial hyperinsulinism type 6 | Autosomal dominant |
| *GM2A* | AB variant GM2 gangliosidosis | Autosomal recessive |
| *GNAS* | McCune-Albright syndrome, glycogen storage disease | Autosomal dominant |
| *GNB1* | T-cell acute lymphoblastic leukemia, somatic mutation | SMu |
| *GNMT* | Glycine N-methyltransferase | Autosomal recessive |
| *GNPTG* | Mucopolysaccharidosis type 3 gamma | Autosomal recessive |
| *GP1BA* | Platelet-type von Willebrand disease | Autosomal dominant |
| *GPHN* | Molybdenum cofactor deficiency | Autosomal recessive |
| *GRN* | GRN-related frontotemporal dementia | Autosomal dominant |
| *GTF2I* | Williams syndrome | Autosomal dominant |
| *GUSB* | Mucopolysaccharidosis type 7 | Autosomal recessive |
| *GYG1* | Glycogen storage disease type XV | Autosomal recessive |
| *GYS1* | Glycogen storage disease type 0 | Autosomal recessive |
| *GYS2* | Glycogen storage disease type 0 | Autosomal recessive |
| *H19* | Beckwith-Wiedemann syndrome | Autosomal dominant |
| *HADHA* | Mitochondrial trifunctional protein deficiency, long-chain L-3-hydroxyacyl-CoA dehydrogenase deficiency | Autosomal recessive |
| *HADHB* | Mitochondrial trifunctional protein deficiency | Autosomal recessive |
| *HAL* | Hypermethioninemia | Autosomal dominant, Autosomal recessive |
| *HAX1* | Severe congenital neutropenia type 3 | Autosomal recessive |
| *HBG2* | Transient neonatal cyanosis | Autosomal dominant |
| *HCFC1* | Methylmalonic acidemia with homocystinuria, cblX type | X-linked recessive |
| *HK1* | Congenital hyperinsulinism | Autosomal recessive |
| *HMGCL* | 3-hydroxy-3-methylglutaryl-CoA lyase deficiency | Autosomal recessive |
| *HNF1A* | Type 1 diabetes mellitus, nonpapillary renal cell carcinoma | Autosomal recessive |
| *HNF4A* | Late-onset type 1 diabetes mellitus | Autosomal dominant |
| *HPD* | Tyrosinemia type 3 | Autosomal recessive |
| *HSD17B10* | HSD10-related mitochondrial disease | X-linked dominant |
| *HSD3B7* | Congenital bile acid synthesis defect type 1 | Autosomal recessive |
| *HSPA9* | Congenital sideroblastic anemia type 4 | Autosomal dominant |
| *HTRA2* | 3-methylglutaconic aciduria type 8 | Autosomal recessive |
| *HYAL1* | Mucopolysaccharidosis type 9 | Autosomal recessive |
| *IFITM5* | Osteogenesis imperfecta type 5 | Autosomal dominant |
| *IGF2* | ? Severe growth retardation and distinctive facial features | Autosomal dominant |
| *IGSF1* | Hypothyroidism with testicular enlargement | X-linked recessive |
| *IL10* | Transplantation-associated susceptibility to graft-versus-host disease | Autosomal dominant |
| *IL10RA* | Inflammatory bowel disease type 28 | Autosomal dominant |
| *IL10RB* | Inflammatory bowel disease type 25 | Autosomal dominant |
| *IL17RD* | Hypogonadotropic hypogonadism type 18 with or without anosmia | Autosomal dominant, Autosomal recessive |
| *IL36RN* | Generalized pustular psoriasis | Autosomal recessive |
| *IL7R* | Autosomal recessive T-negative B-positive-NK-positive severe combined immunodeficiency | Autosomal recessive |
| *INS* | Neonatal persistent diabetes mellitus, insulin-dependent diabetes mellitus type 2 | Autosomal recessive |
| *INSR* | Familial hyperinsulinism-hypoglycemia type 5 | Autosomal dominant |
| *ITGA2B* | Glanzmann thrombasthenia | Autosomal recessive |
| *ITGB3* | Glanzmann thrombasthenia | Autosomal recessive |
| *ITPA* | Early infantile epileptic encephalopathy 35 | Autosomal recessive |
| *IYD* | Hypothyroidism type 4 | Autosomal recessive |
| *JAG1* | Alagille syndrome type 1 | Autosomal dominant |
| *JAK2* | Polycythemia vera | - |
| *JAK3* | JAK3-related severe combined immunodeficiency | Autosomal recessive |
| *JUP* | Naxos disease | Autosomal recessive |
| *KCNC3* | Spinal cerebellar ataxia 13 | Autosomal dominant |
| *KCNH2* | Short QT syndrome type 1 | Autosomal dominant |
| *KCNJ1* | Bartter syndrome type 2 | Autosomal recessive |
| *KCNJ5* | Familial hyperaldosteronism type 3 | Autosomal dominant |
| *KCNQ1* | Familial atrial fibrillation type 3 | Autosomal dominant |
| *KCNQ2* | Benign neonatal seizures type 1 | Autosomal dominant |
| *KCNT1* | Nocturnal frontal lobe epilepsy type 5, early infantile epileptic encephalopathy 14 | Autosomal dominant |
| *KIF5A* | Autosomal dominant spastic paraplegia type 10 | Autosomal dominant |
| *KISS1R* | Hypogonadotropic hypogonadism type 8 | Autosomal recessive |
| *KLF1* | Congenital dyserythropoietic anemia type 4 | Autosomal dominant |
| *KRAS* | Breast cancer, Noonan syndrome type 3 | Autosomal dominant |
| *KRT14* | Localized epidermolysis bullosa simplex | Autosomal dominant |
| *KRT5* | Dowling-Meara epidermolysis bullosa simplex, autosomal recessive epidermolysis bullosa simplex type 1, epidermolysis bullosa simplex with mottled pigmentation, epidermolysis bullosa simplex with migratory circinate erythema, generalized epidermolysis bullosa simplex | Autosomal dominant |
| *LAMA2* | Laminin deficiency congenital muscular dystrophy type 1A | Autosomal recessive |
| *LAMP2* | Danon disease | X-linked dominant |
| *LBR* | Reynolds syndrome | Autosomal dominant |
| *LCT* | Lactose intolerance | Autosomal recessive |
| *LDHA* | Glycogen storage disease type XI | Autosomal recessive |
| *LDLR* | Familial hypercholesterolemia | Autosomal dominant |
| *LDLRAP1* | Autosomal recessive familial hypercholesterolemia | Autosomal recessive |
| *LIG4* | LIG4 syndrome | Unknown |
| *LMBRD1* | Methylmalonic aciduria with homocystinuria, cblF type | Autosomal recessive |
| *LMNA* | Charcot-Marie-Tooth disease type 2B1, Emery-Dreifuss muscular dystrophy type 2, Emery-Dreifuss muscular dystrophy type 3, Hutchinson-Gilford progeria syndrome, Slovenian type heart-hand syndrome, dilated cardiomyopathy with hypogonadotropic hypogonadism, mandibuloacral dysplasia type A, limb-girdle muscular dystrophy type 1B, lethal restrictive dermopathy | Autosomal recessive |
| *LPL* | Familial combined hyperlipidemia | Autosomal dominant |
| *LRP4* | Cenani-Lenz syndactyly syndrome | Autosomal recessive |
| *LYST* | Congenital granule disorder syndrome | Autosomal recessive |
| *MAGED2* | Bartter syndrome type 5 | X-linked recessive |
| *MAGEL2* | Schaaf-Yang syndrome | Autosomal dominant |
| *MANBA* | Beta-mannosidosis | Autosomal recessive |
| *MAP2K1* | Heart-face-skin syndrome type 3 | Autosomal dominant |
| *MAT1A* | Methionine adenosyltransferase I/III deficiency leading to hypermethioninemia | Autosomal dominant, Autosomal recessive |
| *MATN3* | Spondyloepiphyseal dysplasia, maternally inherited 3-related | Autosomal recessive |
| *MC1R* | Melanoma, cutaneous malignant, susceptibility to, 5 | Autosomal recessive |
| *MECP2* | MECP2-related severe neonatal encephalopathy, autism X-linked susceptibility 3, X-linked intellectual disability syndrome type 13, MECP2 duplication syndrome | X-linked recessive |
| *MITF* | Wagner syndrome type 2A | Autosomal dominant |
| *MKKS* | McKusick-Kaufman syndrome | Autosomal recessive |
| *MKRN3* | Central precocious puberty type 2 | Autosomal dominant |
| *MLYCD* | Glutaryl-CoA dehydrogenase deficiency | Autosomal recessive |
| *MOCS2* | Molybdenum cofactor deficiency complementation group B | Autosomal recessive |
| *MPL* | Myelofibrosis, somatic mutation, congenital absence of the radius | - |
| *MRE11A* | Thrombocytopenia, platelet inclusion bodies, with or without nephritis or hearing loss | Autosomal recessive |
| *MT-ATP6* | Capillary malformation-arteriovenous malformation syndrome type 1 | MT |
| *MT-ND3* | Leber hereditary optic neuropathy | MT |
| *MT-TL1* | Leber optic atrophy and dystonia | MT |
| *MUSK* | Mitochondrial myopathy with lactic acidosis and stroke-like episodes | Autosomal recessive |
| *MYBPC3* | Congenital myasthenic syndrome type 9 | Autosomal dominant |
| *MYH7* | Dilated cardiomyopathy 1MM type | Autosomal dominant |
| *MYH9* | Dilated cardiomyopathy 1S type | Autosomal dominant |
| *MYL2* | Giant platelet syndrome with or without nephritis or hearing loss | Autosomal dominant |
| *MYL3* | Familial hypertrophic cardiomyopathy type 10 | Autosomal dominant, Autosomal recessive |
| *MYO9A* | Familial hypertrophic cardiomyopathy type 8 | Autosomal recessive |
| *NADK2* | Congenital myasthenic syndrome | Autosomal recessive |
| *NAGS* | 2,4-dienoyl-CoA reductase deficiency | Autosomal recessive |
| *NBN* | N-acetylaspartate synthetase deficiency | - |
| *NDN* | T-cell acute lymphoblastic leukemia, somatic mutation | IC |
| *NDP* | Prader-Willi syndrome | X-linked recessive |
| *NDUFS4* | Norrie disease, familial exudative vitreoretinopathy type 2 | Autosomal recessive |
| *NEFH* | Mitochondrial complex I deficiency | Autosomal dominant |
| *NEK1* | Charcot-Marie-Tooth disease, axonal type 2CC | DR, Autosomal recessive |
| *NEU1* | Short-rib thoracic dysplasia with or without polydactyly type 6 | Autosomal recessive |
| *NF1* | Sialic acid storage disease | Autosomal dominant |
| *NF2* | Neurofibromatosis type 1 | Autosomal dominant |
| *NFKB1* | Neurofibromatosis type 2 | Autosomal dominant |
| *NHEJ1* | Common variable immunodeficiency type 12 | Unknown |
| *NHLRC1* | Severe combined immunodeficiency with microcephaly, growth retardation, and sensitivity to ionizing radiation | Autosomal recessive |
| *NHP2* | Lafora disease | Autosomal recessive |
| *NKX2-5* | Autosomal recessive congenital ichthyosis type 2 | Autosomal dominant |
| *NOD2* | Non-thyroidal hypothyroidism congenital type 5 | Mu |
| *NOP10* | Growth retardation associated with Crohn's disease | Autosomal recessive |
| *NOTCH3* | Autosomal recessive congenital ichthyosis type 1 | Autosomal dominant |
| *NPAP1* | Autosomal dominant cerebral arteriopathy with subcortical infarcts and leukoencephalopathy type 1 | #N/A |
| *NR5A1* | Prader-Willi syndrome | Autosomal dominant |
| *NRAS* | 46,XX sex reversal type 4, 46,XY sex reversal type 3, spermatogenic failure type 8, premature ovarian failure type 7 | Autosomal dominant |
| *NSMF* | Noonan syndrome type 6 | Autosomal dominant |
| *NUP214* | Hypogonadotropic hypogonadism type 9 with or without anosmia | - |
| *OCRL* | T-cell acute lymphoblastic leukemia, somatic mutation | X-linked recessive |
| *OPA3* | Dent disease type 2, Lowe syndrome | Autosomal recessive |
| *OSTM1* | 3-methylglutaconic aciduria type 3 | Autosomal recessive |
| *P3H1* | Autosomal recessive osteopetrosis type 5 | Autosomal recessive |
| *PARN* | Osteogenesis imperfecta type 8 | Autosomal recessive |
| *PAX6* | Autosomal recessive congenital ichthyosis type 6 | Autosomal dominant |
| *PAX8* | Autosomal dominant eye malformations, coloboma, anterior segment dysgenesis type 5, multiple types, bilateral optic nerve aplasia, iridocorneal endothelial syndrome, central foveal hypoplasia | Autosomal dominant |
| *PC* | Non-thyroidal hypothyroidism congenital type 2 | Autosomal recessive |
| *PCBD1* | Pyruvate carboxylase deficiency | Autosomal recessive |
| *PCDH19* | Tetrahydrobiopterin deficiency type D | X-linked |
| *PCSK9* | Early infantile epileptic encephalopathy 9 | Autosomal dominant |
| *PDHA1* | Familial hypercholesterolemia type 3 | X-linked dominant |
| *PFKM* | E1-alpha ketoglutarate dehydrogenase deficiency | Autosomal recessive |
| *PGAM2* | Glycogen storage disease type VII | Autosomal recessive |
| *PGM1* | Glycogen storage disease type X | Autosomal recessive |
| *PHKA1* | Congenital glycosylation disorder type It | X-linked recessive |
| *PHKB* | Glycogen storage disease type IXd | Autosomal recessive |
| *PHKG1* | Glycogen storage disease type IXb | Autosomal recessive |
| *PHKG2* | Glycogen storage disease | Autosomal recessive |
| *PHOX2B* | Glycogen storage disease type IXc | - |
| *PKD1* | Neuroblastoma susceptibility 2 | Autosomal dominant |
| *PKLR* | Autosomal dominant polycystic kidney disease type 1 | Autosomal dominant |
| *PKP2* | Pyruvate dehydrogenase E1-alpha deficiency | Autosomal dominant |
| *PLEKHM1* | Glycogen storage disease type V | Autosomal recessive |
| *POLG2* | Tetrahydrobiopterin deficiency type C | Autosomal dominant |
| *PON3* | Noonan syndrome type 5 | Autosomal dominant |
| *PPM1K* | Movement disorder, characteristic, with or without seizures | Autosomal recessive |
| *PREPL* | Saposin C deficiency type Gaucher disease | Autosomal recessive |
| *PRKAG2* | Pancreatic agenesis and cerebellar malformation | Autosomal dominant |
| *PRKAG3* | Noonan syndrome type 1 | - |
| *PRODH* | Severe combined immunodeficiency, B-cell and NK-cell positive, T-cell negative, severe combined immunodeficiency, autosomal recessive, T-cell negative, B-cell positive, NK-cell positive | Autosomal recessive |
| *PROKR2* | Muscular dystrophy, lactic acidosis, and sideroblastic anemia type 1 | Autosomal dominant |
| *PRRT2* | Glycogen storage disease type V | Autosomal dominant |
| *PSAP* | Tetrahydrobiopterin deficiency type C | - |
| *PTF1A* | Noonan syndrome type 8 | Autosomal recessive |
| *PTPN11* | Diamond-Blackfan anemia type 7 | Autosomal dominant |
| *PTPRC* | Diamond-Blackfan anemia type 5 | Autosomal recessive |
| *PUS1* | Diamond-Blackfan anemia type 6 |  |
| *PYGM* | Diamond-Blackfan anemia type 9 | Autosomal recessive |
| *QDPR* | Diamond-Blackfan anemia type 4 | Autosomal recessive |
| *RAF1* | Diamond-Blackfan anemia type 1 | Autosomal dominant |
| *RAPSN* | Diamond-Blackfan anemia type 3 | Autosomal recessive |
| *RB1* | Diamond-Blackfan anemia type 10 | Autosomal dominant |
| *RBCK1* | Diamond-Blackfan anemia type 8 | Autosomal recessive |
| *REEP2* | Telomere-related bone marrow failure and/or pulmonary fibrosis type 3 | Autosomal recessive, Autosomal dominant |
| *RET* | Arrhythmogenic right ventricular dysplasia/cardiomyopathy type 2 | Autosomal dominant |
| *RFX5* | Aplastic anemia | Autosomal recessive |
| *RFX6* | Familial hemiplegic migraine type 3, epilepsy, familial focal, with variable foci 2, infantile seizures, familial with paroxysmal choreoathetosis | Autosomal recessive |
| *RFXAP* | Congenital myasthenic syndrome type 16 | Autosomal recessive |
| *RIT1* | Brugada syndrome type 1 | Autosomal dominant |
| *RPL11* | Methionine adenosyltransferase I/III deficiency leading to hypermethioninemia | Autosomal dominant |
| *RPL35A* | Spondyloepiphyseal dysplasia, maternally inherited 3-related | Autosomal dominant |
| *RPL5* | Melanoma, cutaneous malignant, susceptibility to, 5 | Autosomal dominant |
| *RPS10* | MECP2-related severe neonatal encephalopathy, autism X-linked susceptibility 3, X-linked intellectual disability syndrome type 13, MECP2 duplication syndrome | Autosomal dominant |
| *RPS17* | Wagner syndrome type 2A | Autosomal dominant |
| *RPS19* | McKusick-Kaufman syndrome | Autosomal dominant |
| *RPS24* | Central precocious puberty type 2 | Autosomal dominant |
| *RPS26* | Glutaryl-CoA dehydrogenase deficiency | Autosomal dominant |
| *RPS7* | Molybdenum cofactor deficiency complementation group B | Autosomal dominant |
| *RTEL1* | Myelofibrosis, somatic mutation, congenital absence of the radius | Autosomal dominant |
| *RYR2* | Thrombocytopenia, platelet inclusion bodies, with or without nephritis or hearing loss | Autosomal dominant |
| *SBDS* | Capillary malformation-arteriovenous malformation syndrome type 1 | - |
| *SCN1A* | Leber hereditary optic neuropathy | Autosomal dominant |
| *SCN4A* | Leber optic atrophy and dystonia | Autosomal recessive |
| *SCN5A* | Mitochondrial myopathy with lactic acidosis and stroke-like episodes | Autosomal dominant |
| *SCN9A* | Paroxysmal extreme pain disorder, febrile seizures, familial, 7, congenital insensitivity to pain, infantile epileptic encephalopathy 6, erythromelalgia, primary | Autosomal dominant |
| *SDCCAG8* | Bardet-Biedl syndrome type 16 | Autosomal recessive |
| *SEC23B* | Congenital dyserythropoietic anemia type 2 | Autosomal recessive |
| *SECISBP2* | Thyroid hormone metabolism disorder | Autosomal recessive |
| *SEMA3A* | Hypogonadotropic hypogonadism type 16 with or without anosmia | Autosomal dominant |
| *SERPINA1* | Alpha-1 antitrypsin deficiency | Autosomal recessive |
| *SH2B3* | Familial erythrocytosis type 1 | SMu |
| *SH2D1A* | X-linked lymphoproliferative syndrome type 1 | X-linked recessive |
| *SI* | Congenital sucrase-isomaltase deficiency | Autosomal recessive |
| *SLC12A1* | Bartter syndrome type 1 | Autosomal recessive |
| *SLC16A1* | Familial hyperinsulinism type 7 | Autosomal dominant |
| *SLC17A3* | Gout susceptibility type 4 | Autosomal dominant |
| *SLC18A3* | Congenital presynaptic myasthenic syndrome type 21 | Autosomal recessive |
| *SLC19A2* | Thiamine-responsive megaloblastic anemia | Autosomal recessive |
| *SLC25A13* | Adult-onset citrullinemia type II, Heterozygous protein S deficiency, Neonatal citrullinemia type II | Autosomal recessive |
| *SLC25A15* | Hyperornithinemia-hyperammonemia-homocitrullinuria syndrome (HHH syndrome) | Autosomal recessive |
| *SLC25A20* | Carnitine-acylcarnitine translocase deficiency | Autosomal recessive |
| *SLC25A38* | Hereditary spherocytosis type 2 | Autosomal recessive |
| *SLC2A2* | Glycogen storage disease differential diagnosis | Autosomal recessive |
| *SLC34A3* | Hypophosphatemic rickets with hypercalciuria | Autosomal recessive |
| *SLC5A1* | Glucose-galactose malabsorption | Autosomal recessive |
| *SLC5A5* | Thyroid secretion disorder type 1 | Autosomal recessive |
| *SLC5A7* | Distal hereditary motor neuropathy VIIA | Autosomal dominant |
| *SLC7A7* | Lysinuric protein intolerance | Autosomal recessive |
| *SMN2* | Spinal muscular atrophy type 3 | Autosomal recessive |
| *SNAP25* | Congenital myasthenic syndrome type 18 | Autosomal dominant |
| *SNRPN* | Prader-Willi syndrome | IC |
| *SNX10* | Autosomal dominant osteopetrosis type 8 | Autosomal recessive |
| *SOD1* | Amyotrophic lateral sclerosis | Autosomal dominant, Autosomal recessive |
| *SOS1* | Nijmegen syndrome type 4 | Autosomal dominant |
| *SOX10* | Peripheral demyelinating neuropathy, Central myelin formation disorder, VACTERL association and Hirschsprung disease | Autosomal dominant |
| *SPR* | Dihydropyrimidine dehydrogenase deficiency-associated dopamine-responsive dystonia | ?Autosomal dominant, Autosomal recessive |
| *SPTB* | B-cell lymphoproliferative syndrome type 2 | Autosomal dominant |
| *SRD5A2* | Steroid 5-alpha-reductase deficiency | Autosomal recessive |
| *STAT3* | Autosomal dominant hyper-IgE syndrome | Autosomal dominant |
| *STS* | X-linked ichthyosis | X-linked recessive |
| *SUCLA2* | Mitochondrial DNA depletion syndrome type 5 (encephalomyopathic type with methylmalonic aciduria) | Autosomal recessive |
| *SUCLG1* | Mitochondrial DNA depletion syndrome type 9 (encephalomyopathic type with methylmalonic acidemia) | Autosomal recessive |
| *SUMF1* | Multiple sulfatase deficiency | Autosomal recessive |
| *SUOX* | Cystathioninuria | Autosomal recessive |
| *SYT2* | Congenital myasthenic syndrome type 7 | Autosomal dominant |
| *TAL1* | T-cell acute lymphoblastic leukemia with somatic mutation | SMu |
| *TAL2* | T-cell acute lymphoblastic leukemia with somatic mutation | Smu |
| *TAP1* | Naked lymphocyte syndrome type 1 | Autosomal recessive |
| *TAPBP* | Naked lymphocyte syndrome type 1 | Autosomal recessive |
| *TAT* | Tyrosinemia type II | Autosomal recessive |
| *TBX1* | DiGeorge syndrome, Tetralogy of Fallot | Autosomal dominant |
| *TBX20* | Atrial septal defect type 4 | Autosomal dominant |
| *TBX5* | Holt-Oram syndrome | Autosomal dominant |
| *TERC* | Autosomal dominant congenital ichthyosis type 1 | Autosomal dominant |
| *TERT* | Autosomal dominant congenital ichthyosis type 2 | Autosomal dominant, Autosomal recessive |
| *TFAP2B* | Patent ductus arteriosus | Autosomal dominant |
| *TFR2* | Hereditary spherocytosis type 3 | Autosomal recessive |
| *TG* | Thyroid secretion disorder type 3 | Autosomal recessive |
| *TGFB1* | Cystic fibrosis | Autosomal recessive |
| *TGFB3* | Arrhythmogenic right ventricular dysplasia/cardiomyopathy type 1 | Autosomal dominant, |
| *TH* | Tyrosinemia type I | Autosomal recessive |
| *THBD* | Hemolytic uremic syndrome susceptibility type 6 | Autosomal dominant |
| *THPO* | Thrombocytosis type 1 | Autosomal dominant |
| *THRA* | Congenital hypothyroidism type 6 | Autosomal dominant |
| *THRB* | Autosomal dominant generalized resistance to thyroid hormone syndrome | Autosomal dominant |
| *TINF2* | Autosomal dominant congenital ichthyosis type 3 | Autosomal dominant |
| *TMEM38B* | Osteogenesis imperfecta type 14 | Autosomal recessive |
| *TMEM43* | Arrhythmogenic right ventricular dysplasia/cardiomyopathy type 5 | Autosomal dominant |
| *TNFRSF11A* | Early-onset Paget disease of bone type 2 | Autosomal dominant |
| *TNFSF11* | Autosomal dominant osteopetrosis type 2 | Autosomal recessive |
| *TNNC1* | Dilated cardiomyopathy type 1Z | Autosomal dominant |
| *TNNI3* | Dilated cardiomyopathy type 2A | Autosomal recessive |
| *TNNT2* | Dilated cardiomyopathy type 1D | Autosomal dominant |
| *TPM1* | Dilated cardiomyopathy type 1Y | Autosomal dominant |
| *TPO* | Thyroid secretion disorder type 2A | Autosomal recessive |
| *TRH* | Branch chain amino acid metabolism disorder: hyperleucinemia, hyperisoleucinemia, and hypervalinemia | Autosomal recessive |
| *TRHR* | Hypothyroidism | - |
| *TRNT1* | Iron-refractory iron deficiency anemia with B-cell immunodeficiency, periodic fever, and developmental delay |  |
| *TRPM6* | Secondary hypocalcemia and hypomagnesemia | Autosomal recessive |
| *TSC1* | Tuberous sclerosis type 1 | Autosomal dominant |
| *TSC2* | Tuberous sclerosis type 2 | Autosomal dominant |
| *TSHB* | Non-thyroidal congenital hypothyroidism type 4 | Autosomal recessive |
| *TSHR* | Non-thyroidal congenital hypothyroidism type 1, differential diagnosis of glycogen storage disease, and hyperthyroidism | Unknown |
| *TSPAN12* | Familial exudative vitreoretinopathy type 5 | Autosomal dominant |
| *TUPLE1* | DiGeorge syndrome |  |
| *TYK2* | Immunodeficiency 35 | Autosomal recessive |
| *UBE3A* | Angelman syndrome | Autosomal dominant |
| *UBR1* | Branch chain amino acid metabolism disorder: hyperleucinemia, hyperisoleucinemia, and hypervalinemia | Autosomal recessive |
| *UCP2* | Hyperinsulinism | - |
| *UNC13A* | Microcephaly, cortical hyperexcitability, and lethal myopathy | Autosomal recessive |
| *USF1* | Mixed hyperlipidemia type 1 | - |
| *VPS54* | Amyotrophic lateral sclerosis | Autosomal dominant |
| *VWF* | Hemophilia type 1, hemophilia type 3, and hemophilia type 2 | Autosomal dominant、Autosomal dominant, Autosomal recessive |
| *WDPCP* | Bardet-Biedl syndrome type 15 | Autosomal recessive |
| *WDR11* | Hypogonadotropic hypogonadism type 14 | Autosomal dominant |
| *WRAP53* | Autosomal recessive congenital ichthyosis type 3 | Autosomal recessive |
| *XIAP* | X-linked lymphoproliferative syndrome type 2 | X-linked recessive |
| *YARS2* | Myopathy, lactic acidosis, and sideroblastic anemia type 2 |  |
| *ZFPM2* | Fallot tetralogy | Autosomal dominant |
| *ZNF341* | Hyperimmunoglobulin E syndrome |  |

**Supplementary table 2.** Clinical Phenotype of Newborns with Gene Mutations Classified as Pathogenic or Likely Pathogenic Mutations.

| TOP | Gene | Clinical Characteristics | Number of cases | Prevalence among all cases | Prevalence among all participants |
| --- | --- | --- | --- | --- | --- |
| 1 | *UGT1A1* | Neonatal Hyperbilirubinemia, Neonatal Pathological Jaundice | 22 | 23.2% | 1.7% |
| 2 | *FLG* | 1. Eczema or Atopic Dermatitis (Mild)  2. Atopic Dermatitis | 14  1 | 16.5% | 1.2% |
| 3 | *GJB2* | 1. Failed Binaural Hearing Screening  2. Newborn ABO Blood Type Mismatch Hemolytic Disease; Newborn Retinal Hemorrhage | 1  1 | 13.3% | 0.2% |
| 4 | *MEFV* | Patent Foramen Ovale, Patent Ductus Arteriosus; Neonatal Jaundice; Neonatal Infection | 1 | 12.5% | 0.08% |
| 5 | *G6PD* | Glucose-6-Phosphate Dehydrogenase Deficiency; Neonatal Pathological Jaundice; Patent Foramen Ovale, Patent Ductus Arteriosus | 6 | 85.7% | 0.5% |
